# Supplementary material for: Lysosomal trafficking mediated by Arl8b and BORC promotes invasion of cancer cells that survive radiation
Source: Commun Biol. 2020 Oct 27;3:620. doi: 10.1038/s42003-020-01339-9 (PMC7591908; doi:10.1038/s42003-020-01339-9)
Supplement: Supplementary file 1 — Supplementary Information [file 42003_2020_1339_MOESM1_ESM.pdf]

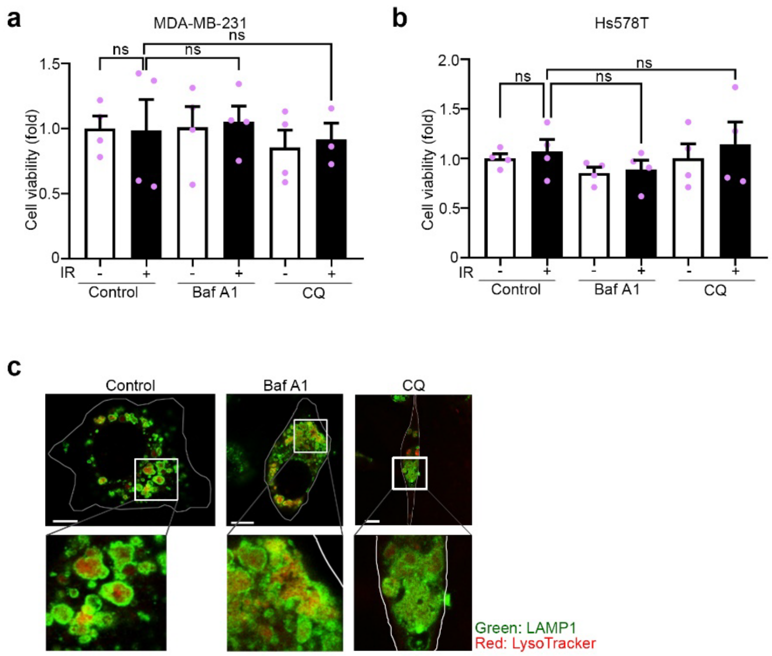

Supplementary Fig. 1 Cell viability, as detected using the Matrigel chemoinvasion assay, is not affected by treatment with lysosome inhibitors. a, b Cell viability of MDA-MB-231 (a) and Hs578T (b) cells after 0 Gy or 4 Gy IR treatment with or without a 12 h treatment with lysosome inhibitors (4 nM Baf A1 or 30 μM CQ) were measured using the Cell Counting Kit-8 (CCK-8) assay. Data were collected from four independent experiments in duplicate and normalized to the control group. Columns, means (n=4); bars, SEMs. ns, not significant. **c** Immunofluorescence images of cells treated for 12 h with lysosome inhibitors. Green, LAMP1; red, LysoTracker Red DND-99. Bar, 10 μm.

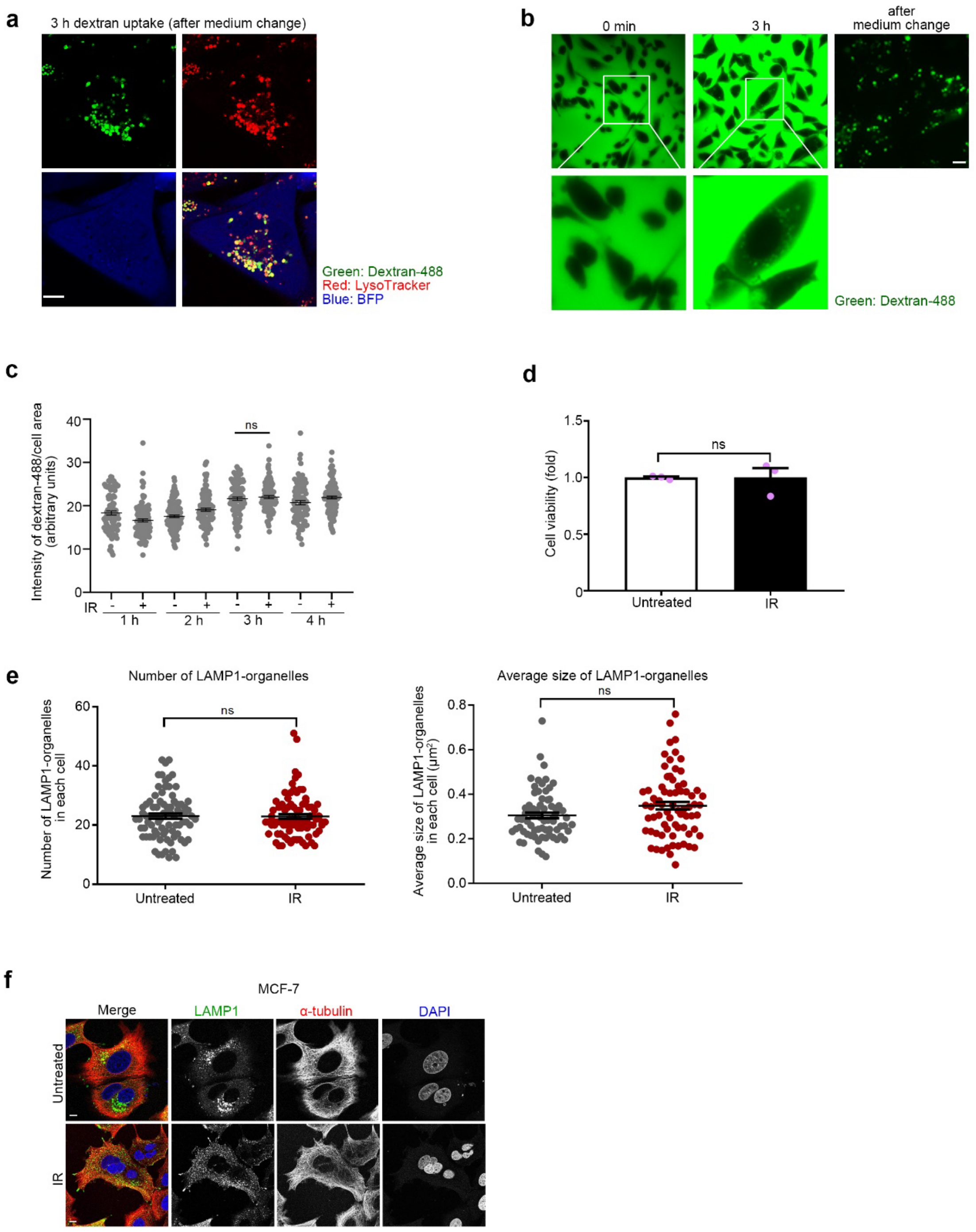

Supplementary Fig. 2 Lysosome exocytosis was quantified by dextran-488 uptake and release. a Immunofluorescence images showing the colocalization of dextran-488 and LysoTracker. Dextran-488 was incubated for 3 h, and the image was captured after a

medium change. Green, dextran-488; red, LysoTracker; blue, blue fluorescent protein (BFP, cell margin). Bar, 10 μm. **b** Representative fluorescent images showing dextran-488 in MDA-MB-231 cells. After 3 h of incubation, the medium was replaced with dextran-488-free medium. After exchange of the media, dextran-488 uptake into the cells was observed. **c** Fluorescence intensities of dextran-488 in MDA-MB-231 cells with or without 4 Gy IR treatment were normalized to each cell area. More than 20 cells per group were assessed in three independent experiments, and the results are shown as a scatter plot. Bars, SEMs. ns, not significant. **d** Cell viability in MDA-MB-231 cells with or without 4 Gy IR treatment during exocytosis assay was measured using the CCK-8 assay. Columns, means (n=3); bars, SEMs. ns, not significant. **e** Number and average size of LAMP1-organelles in each cell of MDA-MB-231 with or without IR treatment. More than 20 cells per group were assessed in three independent experiments, and the results are shown as a scatter plot. Bars, SEMs. ns, not significant. **f** Individual channels of the immunofluorescence images shown in Fig. 1i. Green, LAMP1; red, α-tubulin; blue, DAPI. Bar, 10 μm.

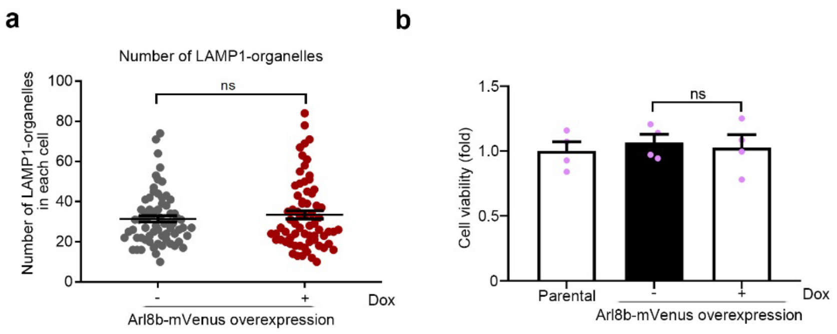

Supplementary Fig. 3 Arl8b overexpression MDA-MB-231 cells. a Arl8b-mVenus overexpression was induced by doxycycline. The number of lysosomes in Arl8b-mVenus-overexpression cells was calculated. More than 20 cells per group were assessed in three independent experiments, and the results are shown as a scatter plot. Bars, SEMs. ns, not significant. **b** Cell viability in MDA-MB-231 cells stably overexpressing Arl8b-mVenus was measured by CCK-8 assay. Dox, doxycycline. Columns, means (n=4); bars, SEMs. ns, not significant.

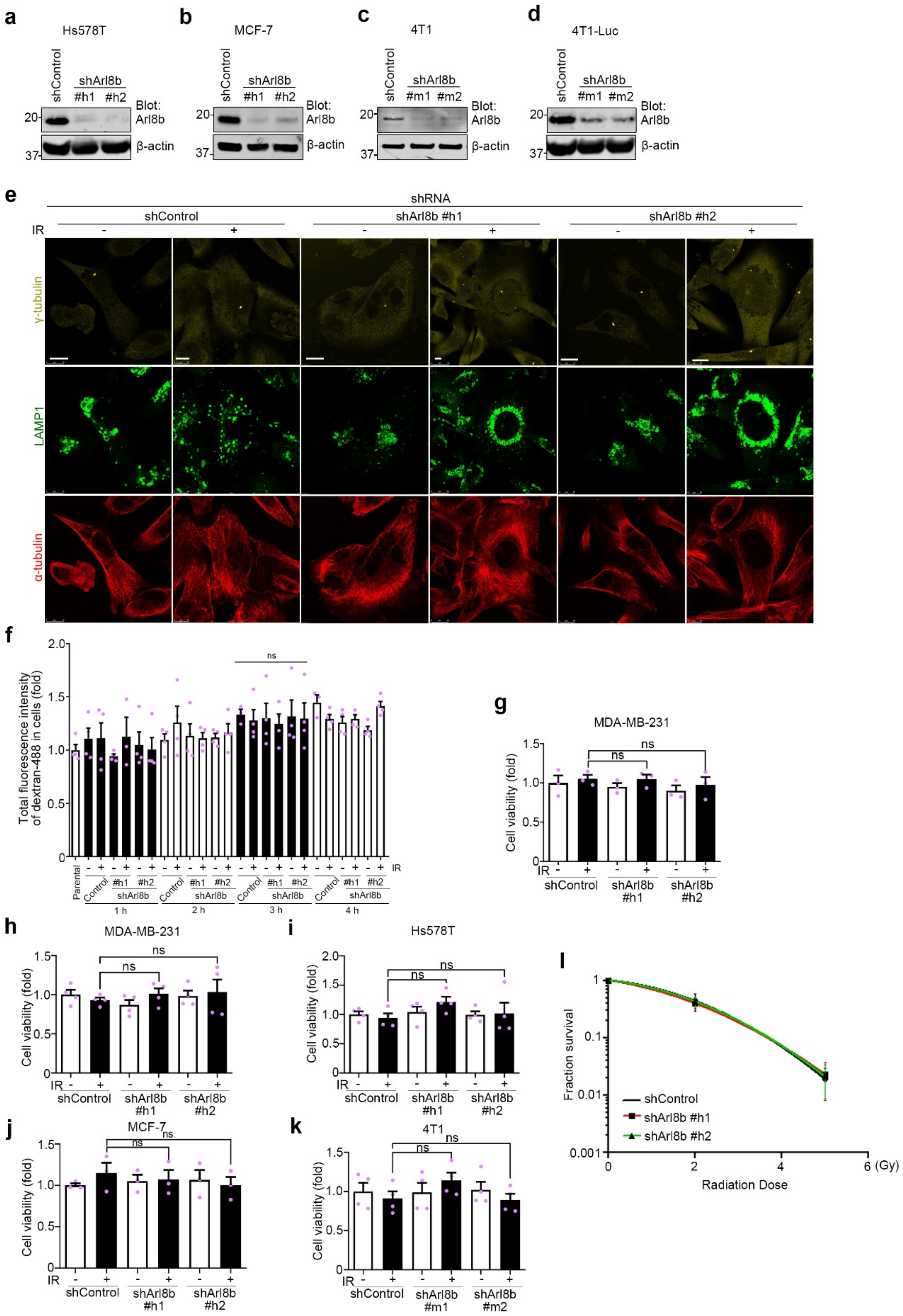
a
Hs578T
shControl
shArl8b
#h1 #h2
Blot: Arl8b
β-actin
b
MCF-7
c
4T1
#m1 #m2
d
4T1-Luc
e
shRNA
shControl
shArl8b #h1
shArl8b #h2
IR
γ-tubulin
LAMP1
α-tubulin
f
Total fluorescence intensity of dextran-488 in cells (fold)
ns
Parental
1 h
2 h
3 h
4 h
g
MDA-MB-231
Cell viability (fold)
h
MDA-MB-231
i
Hs578T
j
MCF-7
k
4T1
l
Fraction survival
shControl
shArl8b #h1
shArl8b #h2
Radiation Dose
6 (Gy)

Supplementary Fig. 4 Effects of Arl8b knockdown on breast cancer cells. a-d Arl8b was knocked down using shRNA human #1 and human #2 in Hs578T cells (a) and MCF-7 cells (b); knocked down using shRNA mouse #1 and mouse #2 in 4T1 cells (c) and 4T1-Luc cells (d). Arl8b protein expression was detected by immunoblotting. **e** Individual channels of the immunofluorescence images shown in Fig. 4b. Yellow, γ-tubulin (centrosome); green, LAMP1; red, α-tubulin. Bar, 10 μm. **f** Knockdown of Arl8b does not affect dextran-488 uptake in cells in an exocytosis assay. The fluorescence intensity of dextran-488 in MDA-MB-231 cells was measured by plate reader after medium change to Dextran-free PBS. Columns, means (n=3); bars, SEMs. ns, not significant. **g** Knockdown of Arl8b does not affect cell viability in an exocytosis assay (Fig. 4e). Cell viability in control or Arl8b-knockdown MDA-MB-231 cells with or without 4 Gy IR treatment during exocytosis assay was measured by CCK-8 assay. Columns, means (n=3); bars, SEMs. ns, not significant. **h-k** Knockdown of Arl8b does not affect cell viability in MDA-MB-231 (h), Hs578T (i) MCF-7 (j) and 4T1 (k) cells during the invasion assay (Fig. 4h-k). Cell viability in control or Arl8b-knockdown MDA-MB-231 cells with or without 4 Gy IR treatment during invasion assay was measured using the CCK-8 assay. Columns, means; bars, SEMs. ns, not significant. **l** Cell survival curves of control or Arl8b-knockdown MDA-MB-231 cells after IR were measured by colony formation assay. Arl8b did not involve in the radiosensitivity of MDA-MB-231 cells. Fractional survival is shown on a log axis, means (n=4); bars, SEMs.

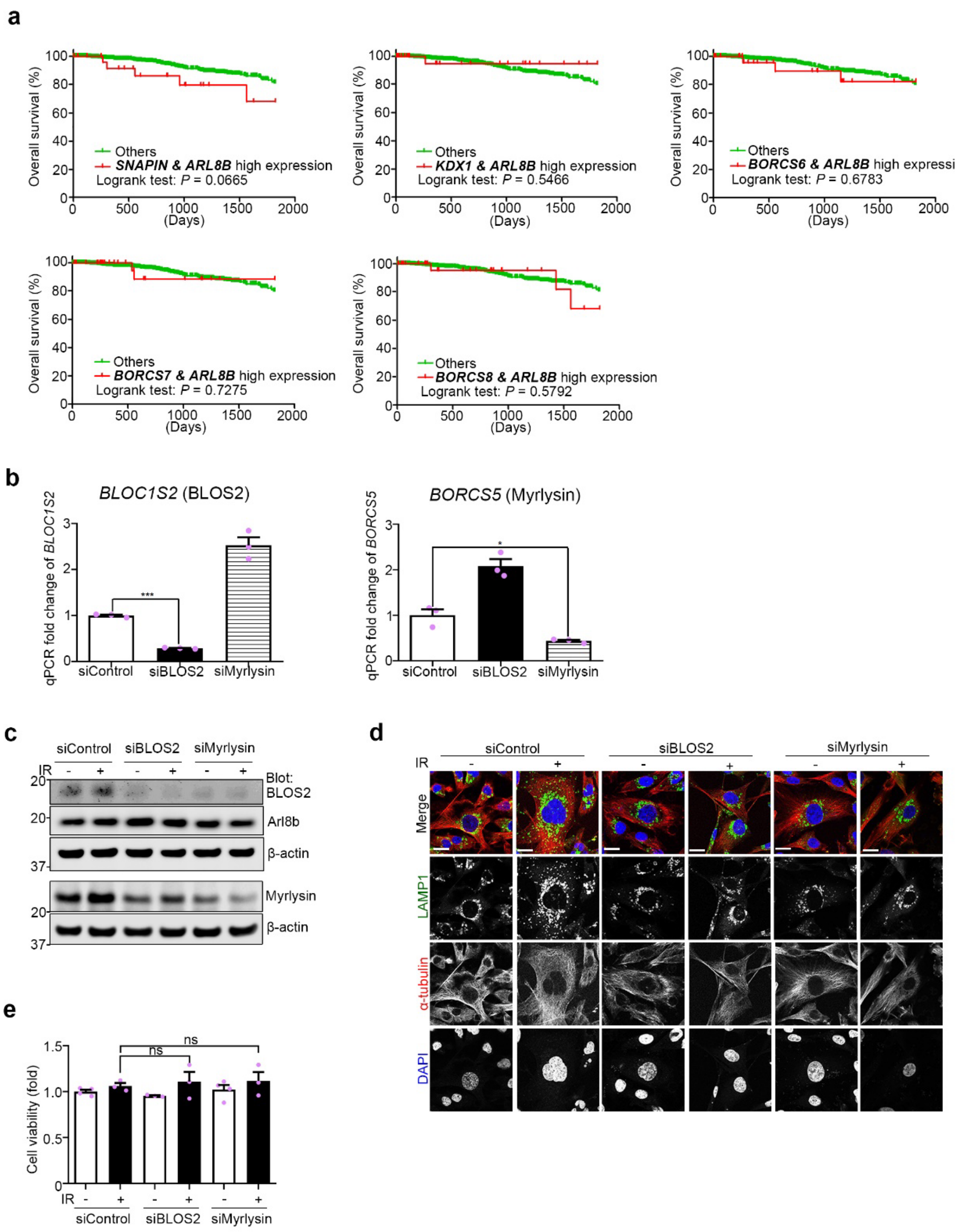

Supplementary Fig. 5 BORC-subunits. a Kaplan-Meier survival analysis based on the expression levels of *ARL8B* and BORC subunits. **b** MDA-MB-231 cells were transfected with siRNAs against BLOS2 or Myrlysin, and changes in expression were confirmed by qPCR. GAPDH was used as an internal control. Columns, means (n=3); bars, SEMs. *, *P*

< 0.01, ***, $P < 0.001$. **c** Protein levels of BLOS2 and Myrlysin were suppressed by knockdown of either Myrlysin or BLOS2. The BLOS2 and Myrlysin protein expression levels were detected by western blotting. **d** Individual channels of the immunofluorescence images shown in Fig. 5f. Green, LAMP1; red, α-tubulin; blue, DAPI. Bar, 20 μm. **e** Cell viability in MDA-MB-231 cells following the siRNA-mediated knockdown of BLOS2 or Myrlysin treated with or without 4 Gy IR used during the invasion assay was measured by CCK-8 assay. Columns, means (n=3); bars, SEMs. ns, not significant.

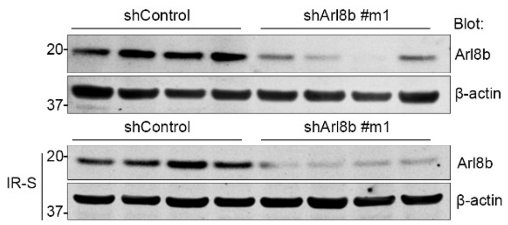

Supplementary Fig. 6 Protein levels of Arl8b in *in vivo* tumors were suppressed by shRNA knockdown. Arl8b protein expression in *in vivo* tumors was detected by immunoblotting.

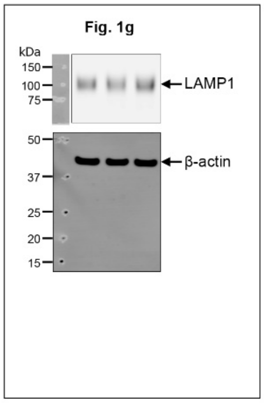
Fig. 1g
kDa
150
100
75
LAMP1
50
β-actin
37
25
20
15

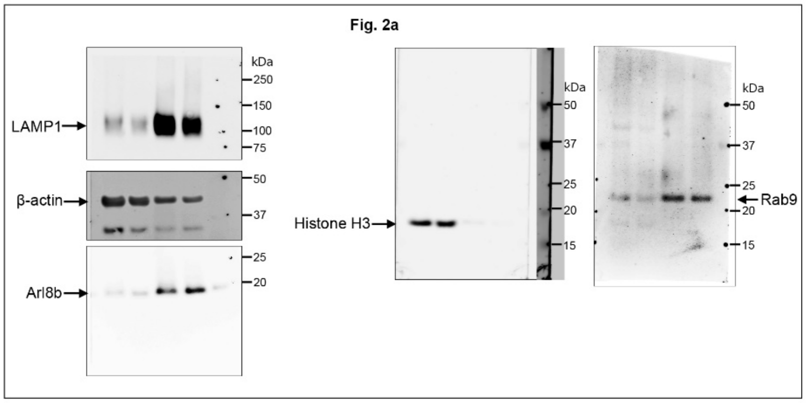
Fig. 2a
kDa
250
150
100
75
LAMP1
50
β-actin
37
25
20
Arl8b
Histone H3
kDa
50
37
25
20
15
kDa
50
37
25
Rab9
20
15

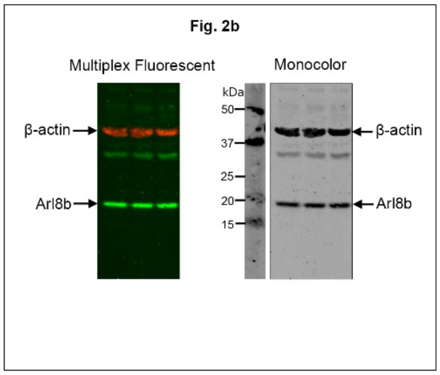
Fig. 2b
Multiplex Fluorescent
Monocolor
β-actin
Arl8b
kDa
50
37
25
20
15
β-actin
Arl8b

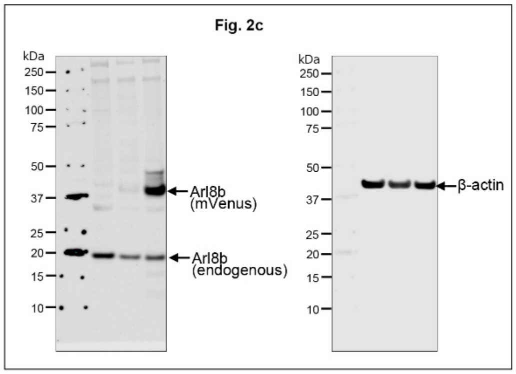
Fig. 2c
kDa
250
150
100
75
50
37
25
20
15
10
Arl8b
(mVenus)
Arl8b
(endogenous)
kDa
250
150
100
75
50
37
25
20
15
10
β-actin

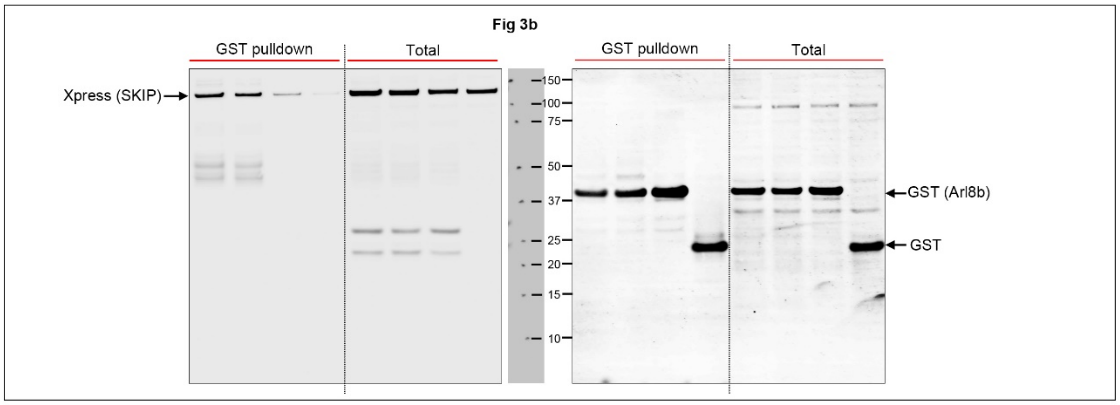
Fig 3b
GST pulldown
Total
Xpress (SKIP)
150
100
75
50
37
25
20
15
10
GST pulldown
Total
GST (Arl8b)
GST

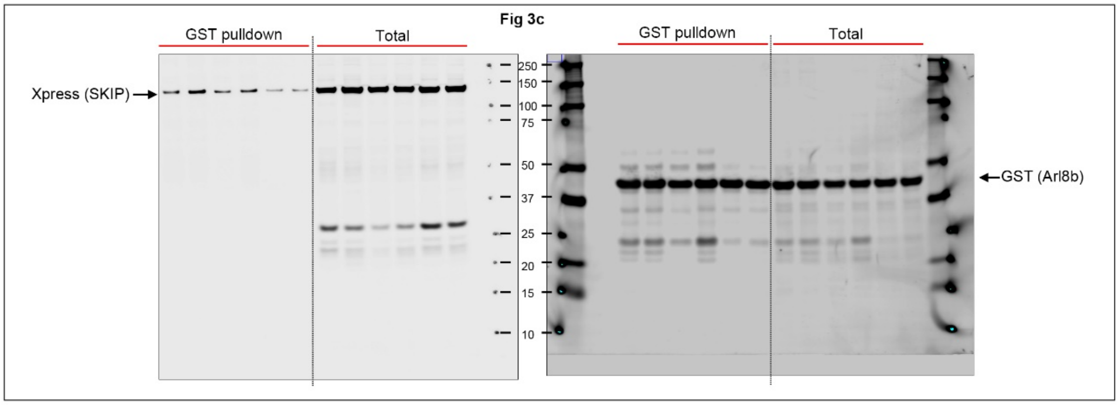
Fig 3c
GST pulldown
Total
Xpress (SKIP)
250
150
100
75
50
37
25
20
15
10
GST pulldown
Total
GST (Arl8b)

Fig.3d

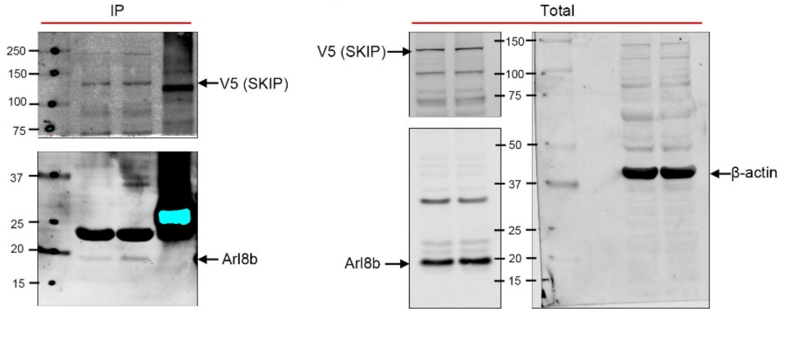

Fig. 4a

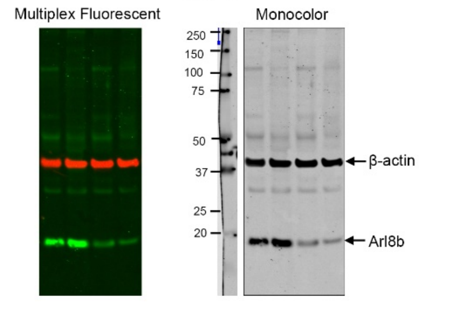

Fig 5d

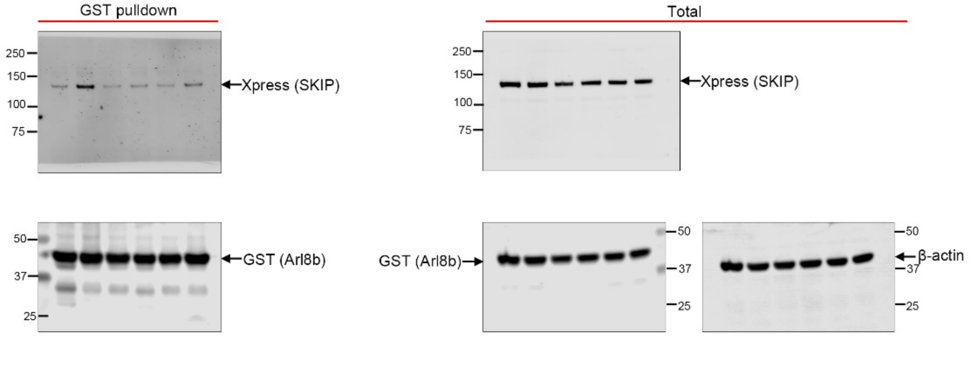

Fig. 6c

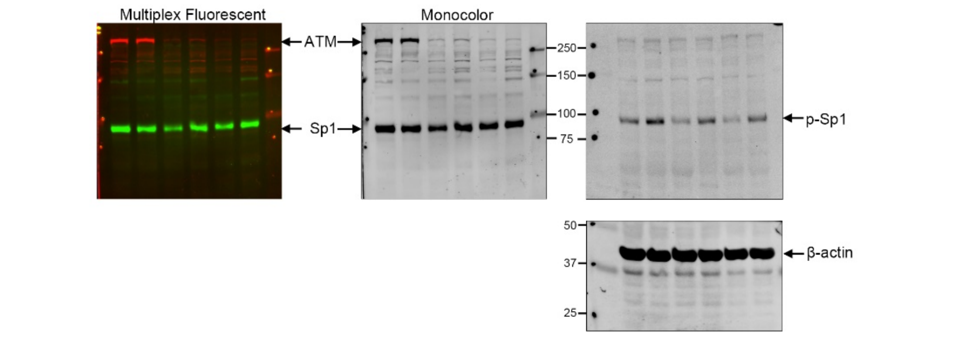

Supplementary Fig. 4a

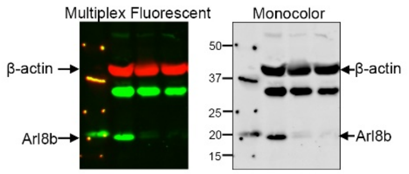

Supplementary Fig. 4b

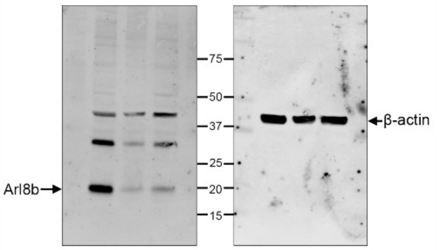

Supplementary Fig. 4c

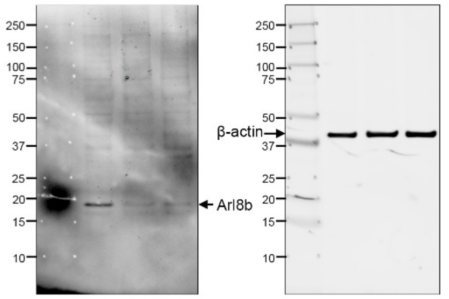

Supplementary Fig. 4d

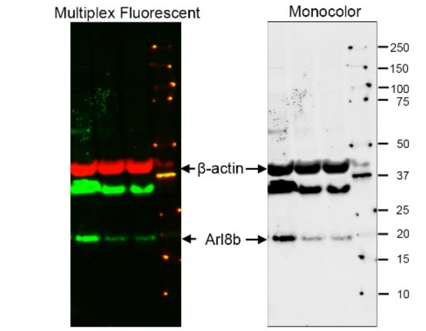

Supplementary Fig. 5c (BLOS2)

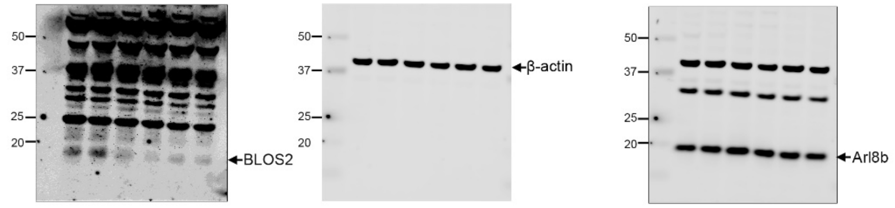

Supplementary Fig. 5c (Myrlysin)

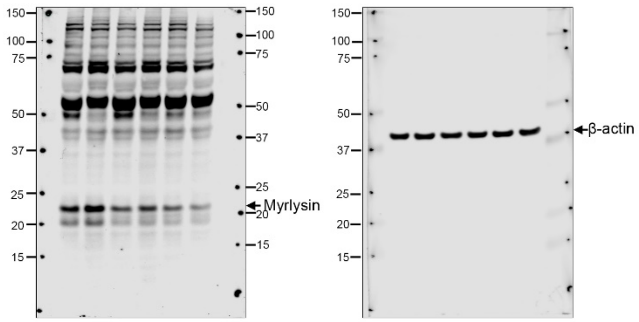

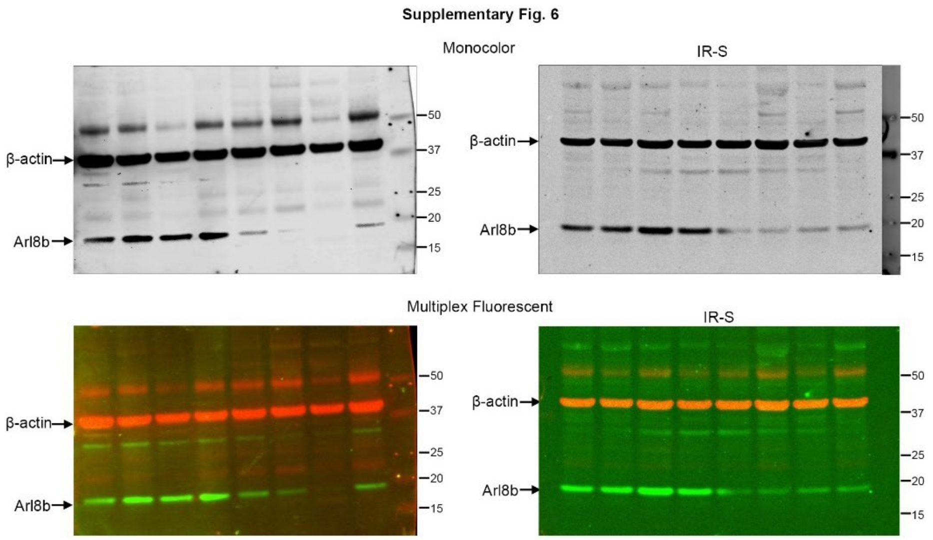

Supplementary Fig. 7 Uncropped scans of western blots.

Supplementary Table 1. List of antibodies

Target	Company	Catalog	Host	Application/ dilution
Arl8b	Proteintech (Rosemont, USA)	13049-1-AP	Rabbit	WB (1:1000)
ATM	Sigma-Aldrich	A1106-25UL	Mouse	WB (1:1000)
BLOC1S2	Abnova (Taipei, Taiwan)	H00282991-M01	Mouse	WB (1:500)
Cathepsin B (D1C7Y)	Cell Signaling Technology	#31718s	Rabbit	IF (1:400)
Cathepsin D	Abcam	ab134169	Rabbit	IF (1:400)
GST tag (EPR4236)	Abcam	ab111947	Rabbit	WB (1:2000) IF (1:400)
HA tag	Medical and Biological Laboratories	M180-3	Mouse	IF (1:400)
Histone H3 (MABI0301)	Medical and Biological Laboratories (Nagoya, Japan)	300-34783	Mouse	WB (1:1000)
Ki67 (D3B5)	Cell Signaling Technology	#12202	Rabbit	IHC (1:400)
LAMP1	Cell Signaling Technology	#9091	Rabbit	WB (1:1000) IF (1:400)
LOH12CR1 (Myrlysin)	Proteintech	17169-1-AP	Rabbit	WB (1:500)
MMP3 (D7f5b)	Cell Signaling Technology	#14351T	Rabbit	IF (1:400)
MT1-MMP (D1E4)	Cell Signaling Technology	#131 30	Rabbit	IF (1:400)
p-Sp1 (Ser101)	Active Motif (Carlsbad, USA)	39758	Rabbit	WB (1:1000)
Rab9	Cell Signaling Technology	#5118	Rabbit	WB (1:1000)
Sp1 (D4C3)	Cell Signaling Technology	#9389	Rabbit	WB (1:1000) ChIP
V5 tag	Thermo Fisher Scientific	R960-25	Mouse	WB (1:1000)
V5 tag (D3H8Q)	Cell Signaling Technology	#13202	Rabbit	WB (1:1000)
Xpress tag	Thermo Fisher Scientific	R910-25	Mouse	WB (1:1000) IF (1:400)
α-tubulin	Cell Signaling Technology	#3873	Mouse	IF (1:400)
β-actin	Sigma-Aldrich	A5441	Mouse	WB (1:5000)
β-actin	Cell Signaling Technology	#4970	Rabbit	WB (1:5000)
γ-tubulin-Alexa Fluor® 647	Abcam (Cambridge, UK)	ab191114	Mouse	IF (1:400)

Supplementary Table 2. List of sequences

shRNA	Sequence (5′-3′)	
Scrambled control	ATCTCGCTTGGGCGAGAGTAAG	
Arl8b #h1	GCTGAAGATGAATATCCCTAA	
Arl8b #h2	AGGTAACGTCACAATAAAGAT	
Arl8b #m1	CGAGGAGTCAATGCAATTGTT	
Arl8b #m2	GCCTCTCGAAATGAACTGCAT	
siRNA	Sequence (5′-3′)	
	Sense	Antisense
Scrambled control	GUUUAUUGACAAGUUAAGAdTdT	UCUUAACUUGUCAAUAAACdTdT
BLOS2	GAUCGGAAUGGUGGAGAACUUdTdT	AAGUUCUCCACCAUUCCGAUCdTdT
Myrlysin	GACCAGAAUGCUUUGGUUAAAdTdT	UUUAACCAAAGCAUUCUGGUCdTdT
Sp1 #1	AAUGAGAACAGCAACAACUCCdTdT	GGAGUUGUUGCUGUUCUCAUUdTdT
Sp1 #2	GAGUCACCCAAUGAGAACAdTdT	UGUUCUCAUUGGGUGACUCdTdT
ATM #1	CAUCUAGAUCGGCAUUCAGdTdT	CUGAAUGCCGAUCUAGAUGdTdT
ATM #2	GCCUCCAAUUCUUCACAGUAAdTdT	UUACUGUGAAGAAUUGGAGGCdTdT
qPCR primers	Sequence (5′-3′)	
	Forward	Reverse
GAPDH	ACAACTTTGGTATCGTGGAAGG	GCCATCACGCCACAGTTTC
18S rRNA	TCGGAACTGAGGCCATGATT	CCTCCGACTTTCGTTCTTGATT
BLOC1S2	AGCTGAGGAAGCAAAGGAGCCT	CCAGGAGCTTATAGTCTTCACTG
BORCS5	GCCGTTGCTTTTGACCAGAATGC	TGCTCGGCATACTTGGCGTATC
SP1	ACGCTTCACACGTTCGGATGAG	TGACAGGTGGTCACTCCTCATG
Negative control for ChIP-qPCR (Chromosome 13 gene desert)	TTTTGACCTACCGTTGCTGA	TCATCTCACCTTGGGTTCACATT
BLOC1S2 promoter (-1237/-1108)	GCTCCTGGACACCCAAAATCT	GGGTGGAGACAGTCAGGGTA
BORCS5 promoter (-669/-518)	GAGAACGCTAGAGCCAGCTAAC	GGGACCTTGGCAGTTATCAACA

Supplementary Table 3. *P*-value

Figure 1a			
t-test		Significant	*P* value
Control	Control IR	**	0.009
Baf A1	Baf A1 IR	ns	0.6033
CQ	CQ IR	ns	0.2994
Control IR	Baf A1 IR	**	0.0011
Control IR	CQ IR	**	0.0022
Figure 1b			
t-test		Significant	*P* value
Control	Control IR	*	0.0100
Baf A1	Baf A1 IR	ns	0.4674
CQ	CQ IR	ns	0.5841
Control IR	Baf A1 IR	**	0.002
Control IR	CQ IR	**	0.0013
Figure 1c			
t-test		Significant	*P* value
Untreated	IR	**	0.0059
Figure 1d			
t-test		Significant	*P* value
Untreated	IR	*	0.0411
Figure 1e			
t-test		Significant	*P* value
Untreated	IR	*	0.0463
Figure 1j			
Mann-Whitney test		Significant	*P* value
Untreated	IR	***	<0.001
Figure 1k			
t-test		Significant	*P* value
Untreated	IR	*	0.0268
Figure 1n			
Mann-Whitney test		Significant	*P* value
Untreated	IR	*	0.0371
Figure 2a			
t-test		Significant	*P* value
Untreated	IR	*	0.0258
Figure 2b			
t-test		Significant	*P* value
0Gy	4Gy	ns	0.4112
0Gy	8Gy	ns	0.6459
Figure 2g			
Mann-Whitney test		Significant	*P* value
Dox -	Dox +	***	<0.001
Figure 2h			

t-test		Significant	*P* value
Dox -	Dox +	*	0.0373
Figure 3c			
t-test		Significant	*P* value
WT	WT IR	**	0.0085
Figure 4d			
Mann-Whitney test		Significant	*P* value
shControl IR	shArl8b #h1 IR	***	<0.001
shControl IR	shArl8b #h2 IR	***	<0.001
Figure 4e			
t-test		Significant	*P* value
shControl IR	shArl8b #h1 IR	*	0.0464
shControl IR	shArl8b #h2 IR	*	0.0375
Figure 4f			
t-test		Significant	*P* value
shControl IR	shArl8b #h1 IR	*	0.0127
shControl IR	shArl8b #h2 IR	*	0.0124
Figure 4g			
t-test		Significant	*P* value
shControl IR	shArl8b #h1 IR	*	0.0101
shControl IR	shArl8b #h2 IR	*	0.0377
Figure 4h			
t-test		Significant	*P* value
shControl	shControl IR	*	0.0150
shControl IR	shArl8b #h1 IR	*	0.0105
shControl IR	shArl8b #h2 IR	*	0.0481
Figure 4i			
t-test		Significant	*P* value
shControl	shControl IR	*	0.0185
shControl IR	shArl8b #h1 IR	**	0.0044
shControl IR	shArl8b #h2 IR	*	0.0323
Figure 4j			
t-test		Significant	*P* value
shControl	shControl IR	*	0.0253
shControl IR	shArl8b #h1 IR	*	0.0397
shControl IR	shArl8b #h2 IR	*	0.0177
Figure 4k			
t-test		Significant	*P* value
shControl	shControl IR	*	0.0225
shControl IR	shArl8b #m1 IR	*	0.0496
shControl IR	shArl8b #m2 IR	*	0.0299
Figure 4m			
Mann-Whitney test		Significant	*P* value
shControl IR	shArl8b #h1 IR	*	0.0113
shControl IR	shArl8b #h2 IR	***	<0.001

Figure 5a			
Logrank test		Significant	*P* value
ARL8B	Others	**	0.0058
BLOC1S1/ARL8B	Others	*	0.0253
BLOC1S2/ARL8B	Others	***	<0.001
BORCS5/ARL8B	Others	*	0.0454
Figure 5b			
Brunner-Munzel test		Significant	*P* value
BLOC1S2/ARL8B	Others	*	0.0326
Figure 5d			
t-test		Significant	*P* value
siControl IR	siBLOS2 IR	*	0.0498
Figure 5h			
Mann-Whitney test		Significant	*P* value
siControl IR	siBLOS2 IR	*	0.0257
siControl IR	siMyrlysin IR	*	0.0304
Figure 5i			
t-test		Significant	*P* value
siControl	siControl IR	*	0.0265
siControl IR	siBLOS2 IR	*	0.0326
siControl IR	siMyrlysin IR	*	0.0204
Figure 6a			
t-test		Significant	*P* value
siControl	siSp1 #1	**	0.0025
siControl	siSp1 #2	**	0.0027
Figure 6b (*BLOC1S2*)			
t-test		Significant	*P* value
siControl IR	siSp1 #1 IR	**	0.0069
siControl IR	siSp1 #2 IR	**	0.0034
Figure 6b (*BORCS5*)			
t-test		Significant	*P* value
siControl IR	siSp1 #1 IR	***	<0.001
siControl IR	siSp1 #2 IR	**	0.0074
Figure 6d (*BLOC1S2*)			
t-test		Significant	*P* value
Anti-Sp1	Anti-Sp1 IR	*	0.0430
Figure 6d (*BORCS5*)			
t-test		Significant	*P* value
Anti-Sp1	Anti-Sp1 IR	*	0.0174
Figure 7c			
t-test		Significant	*P* value
shControl IR-S	shArl8b #m1 IR-S	*	0.0207
Figure 7e			
t-test		Significant	*P* value
shControl IR-S	shArl8b #m1 IR-S	*	0.0321

Figure 7h			
t-test		Significant	*P* value
shControl IR-S	shArl8b #m1 IR-S	***	<0.001
