# Supplementary material for: Lysosomal trafficking mediated by Arl8b and BORC promotes invasion of cancer cells that survive radiation
Source: Commun Biol. 2020 Oct 27;3:620. doi: 10.1038/s42003-020-01339-9 (PMC7591908; doi:10.1038/s42003-020-01339-9)
Supplement: Supplementary file 3 — Description of Additional Supplementary Files [file 42003_2020_1339_MOESM3_ESM.pdf]

Description of Additional Supplementary Files

File Name: Supplementary Data 1
Description: Source data of main figures
