# Supplementary material for: Lysosomal trafficking mediated by Arl8b and BORC promotes invasion of cancer cells that survive radiation
Source: Commun Biol. 2020 Oct 27;3:620. doi: 10.1038/s42003-020-01339-9 (PMC7591908; doi:10.1038/s42003-020-01339-9)
Supplement: Supplementary file 5 — Reporting Summary [file 42003_2020_1339_MOESM5_ESM.pdf]

nature research

Corresponding author(s): Jin-Min Nam
Yasuhito Onodera

Last updated by author(s): Aug 26, 2020

Reporting Summary

Nature Research wishes to improve the reproducibility of the work that we publish. This form provides structure for consistency and transparency in reporting. For further information on Nature Research policies, seeAuthors & Referees and theEditorial Policy Checklist .

Statistics

For all statistical analyses, confirm that the following items are present in the figure legend, table legend, main text, or Methods section.

n/a	Confirmed	
☐	☒	The exact sample size (*n*) for each experimental group/condition, given as a discrete number and unit of measurement
☐	☒	A statement on whether measurements were taken from distinct samples or whether the same sample was measured repeatedly
☐	☒	The statistical test(s) used AND whether they are one- or two-sided<br>*Only common tests should be described solely by name; describe more complex techniques in the Methods section.*
☐	☒	A description of all covariates tested
☐	☒	A description of any assumptions or corrections, such as tests of normality and adjustment for multiple comparisons
☐	☒	A full description of the statistical parameters including central tendency (e.g. means) or other basic estimates (e.g. regression coefficient) AND variation (e.g. standard deviation) or associated estimates of uncertainty (e.g. confidence intervals)
☐	☒	For null hypothesis testing, the test statistic (e.g. *F*, *t*, *r*) with confidence intervals, effect sizes, degrees of freedom and *P* value noted<br>*Give P values as exact values whenever suitable.*
☐	☒	For Bayesian analysis, information on the choice of priors and Markov chain Monte Carlo settings
☐	☒	For hierarchical and complex designs, identification of the appropriate level for tests and full reporting of outcomes
☐	☒	Estimates of effect sizes (e.g. Cohen's *d*, Pearson's *r*), indicating how they were calculated

Our web collection on statistics for biologists contains articles on many of the points above.

Software and code

Policy information about availability of computer code

Data collection	No software and code applied in the study.
Data analysis	No software and code applied in the study.

For manuscripts utilizing custom algorithms or software that are central to the research but not yet described in published literature, software must be made available to editors/reviewers. We strongly encourage code deposition in a community repository (e.g. GitHub). See the Nature Research guidelines for submitting code & software for further information.

Data

Policy information about availability of data

All manuscripts must include a data availability statement. This statement should provide the following information, where applicable:

- Accession codes, unique identifiers, or web links for publicly available datasets
- A list of figures that have associated raw data
- A description of any restrictions on data availability

Data availability statement: Source data for figures are provided in Supplementary data 1. Uncropped scans of western blot are shown in Supplementary Fig. 7. Microarray data was deposited in the Gene Expression Omnibus (GEO) under accession number GSE155785. RNA-seq and clinical data from breast cancer patients (Project ID: TCGA-BRCA) was obtained at the TCGA web site (URL: http://cancergenome.nih.gov/).

Field-specific reporting

Please select the one below that is the best fit for your research. If you are not sure, read the appropriate sections before making your selection.

Life sciences study design

All studies must disclose on these points even when the disclosure is negative.

Sample size	No statistical method was used to predetermine sample size.
Data exclusions	None of the samples were excluded from the experiment, unless there were clear technical mistakes.
Replication	All the in vitro experiments were repeated at least three times to verify the reproducibility of the experimental findings.
Randomization	Samples (cultured cells, mice) were random allocation into experimental groups and performed in essentially the same manner.
Blinding	Investigators were not blinded during the experiments and outcome assessment, but had no pre-conceptions.

Reporting for specific materials, systems and methods

We require information from authors about some types of materials, experimental systems and methods used in many studies. Here, indicate whether each material, system or method listed is relevant to your study. If you are not sure if a list item applies to your research, read the appropriate section before selecting a response.

Materials & experimental systems

n/a	Involved in the study	
☐	☒	Antibodies
☐	☒	Eukaryotic cell lines
☒	☐	Palaeontology
☐	☒	Animals and other organisms
☒	☐	Human research participants
☒	☐	Clinical data

Methods

n/a	Involved in the study	
☒	☐	ChIP-seq
☐	☒	Flow cytometry
☒	☐	MRI-based neuroimaging

Antibodies

Antibodies used	Please refer to Supplementary Table 1.
Validation	Please refer to Supplementary Table 1.

Eukaryotic cell lines

Policy information about cell lines

Cell line source(s)	All cell lines were purchased from American Type Culture Collection (ATCC) and expanded following their instructions.
Authentication	All cell lines were purchased from ATCC and authenticated by ATCC.
Mycoplasma contamination	Cell lines were tested for mycoplasma contamination with the staining with DAPI or Hoechst 33342 before whole experiments.
Commonly misidentified lines (See ICLAC register)	No misidentified cell lines were used in the study.

Animals and other organisms

Policy information about studies involving animals; ARRIVE guidelines recommended for reporting animal research

Laboratory animals	6-week-old female BALB/c-nu/nu mice purchased from Hokudo Co.,Ltd were used in the study.
Wild animals	No wild animals were involved in the study.
Field-collected samples	No field-collected samples were involved in the study.
Ethics oversight	All animal procedures were approved by the Institutional Animal Care and Use Committee of Hokkaido University (# 16-0137).

Note that full information on the approval of the study protocol must also be provided in the manuscript.

Flow Cytometry

Plots

Confirm that:

- [x] The axis labels state the marker and fluorochrome used (e.g. CD4-FITC).
- [x] The axis scales are clearly visible. Include numbers along axes only for bottom left plot of group (a 'group' is an analysis of identical markers).
- [x] All plots are contour plots with outliers or pseudocolor plots.
- [x] A numerical value for number of cells or percentage (with statistics) is provided.

Methodology

Sample preparation	Please refer to the method "Flow cytometry"
Instrument	A FACSAria III flow cytometer (BD Biosciences) was used in the study.
Software	BD FACSDiva Software (BD Biosciences)
Cell population abundance	10,000 per samples were analyzed.
Gating strategy	No gating was used in the study. Mean fluorescence intensity was used in the study.

- [x] Tick this box to confirm that a figure exemplifying the gating strategy is provided in the Supplementary Information.
